# Metals in fungal virulence

**DOI:** 10.1093/femsre/fux050

**Published:** 2017-10-23

**Authors:** Franziska Gerwien, Volha Skrahina, Lydia Kasper, Bernhard Hube, Sascha Brunke

**Affiliations:** Department Microbial Pathogenicity Mechanisms, Leibniz Institute for Natural Product Research and Infection Biology– Hans Knoell Institute, 07745 Jena, Germany

**Keywords:** transition metals, pathogenic fungi, nutritional immunity, metal homeostasis, host–pathogen interactions, regulatory networks

## Abstract

Metals are essential for life, and they play a central role in the struggle between infecting microbes and their hosts. In fact, an important aspect of microbial pathogenesis is the ‘nutritional immunity’, in which metals are actively restricted (or, in an extended definition of the term, locally enriched) by the host to hinder microbial growth and virulence. Consequently, fungi have evolved often complex regulatory networks, uptake and detoxification systems for essential metals such as iron, zinc, copper, nickel and manganese. These systems often differ fundamentally from their bacterial counterparts, but even within the fungal pathogens we can find common and unique solutions to maintain metal homeostasis. Thus, we here compare the common and species-specific mechanisms used for different metals among different fungal species—focusing on important human pathogens such as *Candida albicans*, *Aspergillus fumigatus* or *Cryptococcus neoformans*, but also looking at model fungi such as *Saccharomyces cerevisiae* or *A. nidulans* as well-studied examples for the underlying principles. These direct comparisons of our current knowledge reveal that we have a good understanding how model fungal pathogens take up iron or zinc, but that much is still to learn about other metals and specific adaptations of individual species—not the least to exploit this knowledge for new antifungal strategies.

## Teaser

Pathogenic fungi require metals to survive and cause disease in the host. Their complex regulatory, uptake and detoxification systems are often uniquely adapted to conditions *in vivo*. This review compares and contrasts metal homeostasis mechanisms of human fungal pathogens.

## INTRODUCTION

Fungi are frequently underestimated as causes of disease and death worldwide—by the public, by health practitioners, and even by national and global health organizations (Brown *et al*. 2012). Because of their often high mortality rates, infections with invasive fungi from genera as diverse as *Candida, Aspergillus*, *Cryptococcus, Histoplasma, Paracoccidioides* or *Blastomyces* are responsible for about one and a half million deaths per year (Brown *et al*. 2012), and non-fatal infections will affect most people at least once in their lifetime, with correspondingly high costs for healthcare systems worldwide. The search for fungal virulence factors and thus potential new drug targets in these eukaryotic pathogens is therefore all the more important.

Metals play a surprisingly central role in infection processes, as they serve as cofactors in a multitude of enzymes—including many with direct and indirect roles in virulence, such as metal-dependent superoxide dismutases (SODs), metalloproteases or melanin-producing laccases. Especially the first-row transition metals—manganese (Mn), iron (Fe), cobalt (Co), nickel (Ni) and copper (Cu)—provide the necessary redox and catalytic activity for many important biological processes. Their ionization energies increase slowly both over the row and for subsequent ionization events in the same metal. In the case of first-row transition metals, this is due to the shielding effect of their 3d-electrons on the 4s-electrons, and these are first lost during ionization. In fact, all these transition metals thus have a stable +2 oxidation state (lacking the 4s-electrons) and generally many additional stable states (up to seven in the case of Mn), which allows them to readily change their oxidation states in biological reactions. Zinc (Zn), with its single oxidation state (+2) and its filled d-orbital, is a notable exception, but nonetheless plays important roles especially in eukaryotic gene regulation.

The host is similarly dependent on metals, and should theoretically present a near optimal, metal-rich environment for infecting microbes. However, this is counterintuitively not the case, a fact that helps our intact immune system to fend off pathogenic fungi and bacteria. This is due to a process aptly named ‘nutritional immunity’, where the host actively sabotages and counteracts metal uptake by microorganisms (Weinberg 1975) and to make matters worse—as seen from the pathogen's side—can also fight invaders by deploying toxic levels of certain metals (Hood and Skaar 2012). Iron, copper and manganese, for example, are intrinsically toxic via Fenton chemistry (Fenton 1894), the metal-catalyzed generation of oxygen radical species from hydrogen peroxide, which at high metal concentrations results in oxidative damage to the microbes (Higson, Kohen and Chevion 1988; Touati 2000). Furthermore, many of the common biological metals have similar divalent cation properties in binding ligands, but strikingly different catalytic functions. Mismetallation, i.e. the replacement of an enzyme's metal cofactor by a different metal by host-induced metal excess and oxidative stress (reviewed in Imlay 2014), could thus inhibit the function of microbial enzymes that require defined metals as cofactors (Macomber and Imlay 2009; McDevitt *et al*. 2011; Veyrier *et al*. 2011). Consequently, the pathogens must keep these essential metals within strict homeostatic boundaries even when moving through rapidly changing metal microenvironments within the host. Finally, in biologically relevant pH ranges, these metals are frequently more soluble under acidic conditions, which results in often pH-dependent systems of metal homeostasis, many of which are described below.

Many of the metal conditions in microbial organisms still reflect the environment that we envision to have existed during the emergence of life. Then, iron was mainly present in its ferrous form (Fe^2+^)—due to the anoxic environment, which also led to copper and other soft metals to be trapped away in sulfide minerals. Especially eukaryotes, like fungi, later learned to include zinc and, to a certain extent, copper into the spectrum of biologically useful metals. Still, the profound differences between the evolutionary inherited patterns of metal use and the modern lower availability of iron (mostly ferric (Fe^3+^) rather than ferrous, due to the newly oxic conditions), and the relative abundance of soft metals, like copper, presents a continuing challenge to microbes, which nonetheless may have ‘trained’ the microorganisms to better deal with the metal-based nutritional immunity of mammals.

In fact, pathogenic fungi have developed often complex and advanced detection and signaling networks to upregulate the import of specific metals in times of need. Frequently, biological processes that rely on these metals are downregulated by dedicated regulators, reducing the consumption and liberating the bound metal. Under metal excess, often (but not always) a different regulator stops the expression of importers and initiates the sequestration of surplus metal to special proteins like metallothioneins (MTs) or to the vacuole, which serves as an overflow basin and emergency reservoir for many different metals. Many transporters have evolved that allow the transport of the charged metal ions over the plasma or vacuolar membranes, but unspecific transport of several metals by the same transporter is not uncommon—bringing with it the danger of the loss of full control over the metals that enter the cell and possibly leaving the microbe vulnerable to metal toxicity (Liu *et al*. 1997; Li and Kaplan 1998; Viau *et al*. 2012; Caetano *et al*. 2015).

Excellent recent reviews exist on many aspects of bacterial metal use, and among those we highly recommend (Palmer and Skaar 2016) for readers interested in non-fungal systems. On the topic of nutritional immunity, we recommend (Hood and Skaar 2012) for an outstanding overview of metal-related bacteria–host interactions, and (Crawford and Wilson 2015) for a view on common fungal pathogens. For an in-depth view on individual metals and their role in microbial pathogenesis, we refer the reader to Garcia-Santamarina and Thiele (2015) for copper, and for iron to Ganz and Nemeth (2015) and Soares and Weiss (2015) for a host view and Bairwa, Hee Jung and Kronstad (2017) for the fungal side.

In this review, we compile and compare strategies that fungi employ to obtain metals during pathogenesis, and we provide examples for different homeostatic mechanisms, and how they connect to fungal virulence. To this end, we summarize here the basic principles of homeostatic regulation in pathogenic fungi for iron, zinc, copper and manganese—metals for which a sufficiently large body of literature exists. The direct comparisons of known mechanisms among fungi will, we hope, allow the reader to discover common principles and identify open questions in order to complete our picture of the role of metals in fungal infections.

## IRON

Most texts on microbial metal homeostasis start with a focus on iron. This is for good reason, as iron is the most abundant of the trace metals in organisms and arguably the one with the most diverse roles in cellular processes. These include central metabolic pathways such as oxygen transport, the tricarboxylic acid (TCA) cycle or electron transport chains, mostly via incorporation of iron or the iron-containing prosthetic group heme into the active centers of key enzymes. For these reasons, iron is an essential metal in nearly all organisms (*Borrelia burgdorferi*, the causative agent of Lyme disease, is one of the rare and notable exceptions; Posey and Gherardini 2000). While the ubiquity of iron is related to its chemical redox properties, namely the capacity to readily switch between the ferric and the ferrous form, this same quality is also at the root of the problems that can be caused by iron in many biological systems. For instance Fe^3+^, the prevalent form under aerobic conditions, is essentially insoluble in water and hence inaccessible to most microbes. Fe^2+^ in contrast is much more soluble, but at the same time more prone to elicit iron-induced toxicity mediated by the formation of radicals via the Fenton reaction. Additionally, iron, similar to copper, has a high affinity to replace other metals in enzymatic reactive centers, a mismetallation that usually results in a disruption of the enzymatic function (Vance and Miller 1998; Martin and Imlay 2011).

Accordingly, vertebrates and microorganisms alike have developed sophisticated strategies to ensure solubility, distribution and steady supply of iron while keeping its homeostatic levels sufficiently low to prevent toxicity. In vertebrates, this includes the almost complete binding of iron via a plethora of transport and storage proteins, such as hemoglobin, transferrin, lactoferrin and ferritin (reviewed in Wang and Pantopoulos 2011). During infection, microbial access to iron (and other metals) is actively restricted even further by nutritional immunity mechanisms (Weinberg 1975). This occurs at the systemic level by hepcidin-induced reduction of circulating iron (Nemeth *et al*. 2004) and at the tissue level by the active redistribution of iron away from sites of infection (Potrykus *et al*. 2013). In these processes, iron is shuttled to intracellular stores to keep it out of reach of invading pathogens—predominantly in macrophages, which also act as natural heme recycling sites via phagocytosis of senescent erythrocytes (reviewed in Wang and Pantopoulos 2011).

However, a range of microbial pathogens have adopted an intracellular lifestyle and use macrophages as hiding places from the immune system, or even as a source of nutrients and metals for their own growth. This includes many pathogenic fungi such as the dimorphic ascomycete *Histoplasma capsulatum* (Newman *et al*. 1994; Hwang *et al*. 2008), the basidiomycete *Cryptococcus neoformans* (Levitz *et al*. 1997), the yeast-like ascomycete *Candida glabrata* (Nevitt and Thiele 2011; Seider *et al*. 2014) and other dimorphic ascomycetes e.g. *Paracoccidioides brasiliensis* (Cano *et al*. 1994) or *Blastomyces dermatitidis* (Sterkel *et al*. 2015). All these species are able to survive phagocytosis and replicate inside macrophages, and they use diverse strategies in order to exploit the intracellular iron stores of macrophages, not all of which have yet been elucidated (Hilty, Smulian and Newman 2008, 2011; Nevitt and Thiele 2011; Hu *et al*. 2015).

### Iron homeostasis and uptake

**Figure 1.**
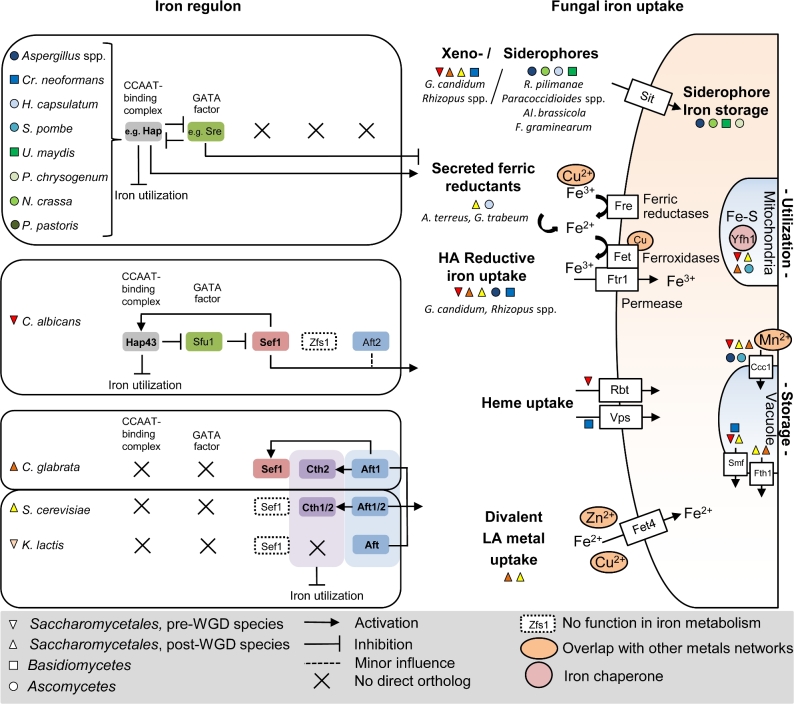
Fungal iron homeostasis. Regulation of iron homeostasis (left panel side) is shown for different fungal species (species is color coded, shape defines phylogenetic ancestry according to Gabaldon *et al*. 2013). Major transcription factors upregulated during iron starvation to initiate fungal iron uptake (right panel side) are written in bold. Functional orthologs are color shaded and aligned vertically, X indicates lack of ortholog and a white box with dashed borders indicates that an ortholog is present but not involved in iron homeostasis. HA, high affinity; LA, low affinity.

Pathogens have evolved elaborate systems to acquire iron from their environment (Fig. 1). A common theme in iron uptake is the utilization of siderophores, a heterogeneous class of small molecules, which are secreted by bacteria and fungi to bind extracellular ferric iron with extremely high affinity. This is achieved by coordinating Fe^3+^ by normally six oxygen ligands per molecule in an octahedral geometry, although siderophores with less donor atoms per molecule can bind in stochiometries different from 1:1 or use water as an additional oxygen donor. Siderophore–iron complexes are then either taken up directly or they deliver their precious load to receptors of the microbe's surface for uptake via specific transporters (reviewed for fungi in Haas, Eisendle and Turgeon 2008). Like in bacteria, many different classes of fungal siderophores are known, such as the most commonly produced hydroxamates [triacetylfusarinine C (Charlang *et al*. 1981; Oide *et al*. 2006; Schrettl *et al*. 2007), coprogens (Matzanke *et al*. 1987), ferrichromes (Neilands 1952), rhodotorulic acid (Muller, Barclay and Raymond 1985)], polycarboxylates produced by zygomycetes (Thieken and Winkelmann 1992) and phenolates-catecholates, which are present in wood-rotting fungi (Fekete, Chandhoke and Jellison 1989). Some fungal siderophores have highly specialized roles: *Aspergillus fumigatus* and *A. nidulans* ferricrocins, for example, are found inside the fungus rather than being secreted, and are involved in intracellular iron homeostasis and storage (Eisendle *et al*. 2006; Schrettl *et al*. 2007; Gsaller *et al*. 2012). Similarly, ferrichromes of the plant-pathogenic fungi *Ustilago sphaerogena* and *U. maydis* can be secreted or store iron intracellularly (Ecker, Lancaster and Emery 1982; Budde and Leong 1989). Importantly, Fe^3+^ bound to siderophores, due to their strongly negative redox potential, is not readily reduced to Fe^2+^ and hence will not generate hydroxyl radicals (Cornish and Page 1998). By this mechanism, intracellular siderophores can help to protect microbes from the toxic effects of iron (Eisendle *et al*. 2006).

Overall, siderophore producers are widespread in the fungal kingdom and include animal and human pathogens such as *Aspergillus* spp. (Zähner *et al*. 1963; Nilius and Farmer 1990; Gressler *et al*. 2015), *H. capsulatum* (Howard *et al*. 2000), *Rhodotorula pilimanae* (Carrano and Raymond 1978), *Neurospora crassa* (Horowitz *et al*. 1976), *Paracoccidioides* spp. (Silva-Bailao *et al*. 2014) and the plant pathogens *U. maydis* (Budde and Leong 1989) and *Alternaria brassicicola* (Oide *et al*. 2006), among many others. In fact, siderophores are essential for the virulence of most fungal pathogens producing them. Deletion mutants lacking siderophore synthesis genes show severe virulence defects in *A. fumigatus* (Schrettl *et al*. 2004; Hissen *et al*. 2005), and also in *H. capsulatum* (Hwang *et al*. 2008). Consequently, the host has been shown to sequester fungal (and bacterial) siderophores via siderocalins, special siderophore-binding lipocalins (Goetz *et al*. 2002; Leal *et al*. 2013). Notably, the cellular energy cost to sustain siderophore synthesis is rather high for the microbe. Hence, biosynthesis is generally tightly controlled and activated solely upon significant iron shortage (Mei, Budde and Leong 1993; Oberegger *et al*. 2001). In addition, many fungal species, including *C. albicans*, *C. glabrata* or *Saccharomyces cerevisiae*, as well as *Cr. neoformans, Geotrichum candidum* and *Rhizopus* spp., lack the key enzyme L-ornithine N5-oxygenase (Sid1/SidA), which is needed for the initiation of hydroxamate siderophore biosynthesis, and they thus do not produce their own siderophores (reviewed in Haas, Eisendle and Turgeon 2008). Controversially, siderophore production was reported for *C. albicans* (Ismail, Bedell and Lupan 1985), but no putative biosynthesis genes were subsequently found in the genome.

Lacking their own biosynthetic machinery, these species often rely on xenosiderophores, i.e. siderophores produced by other fungi or bacteria. Dedicated xenosiderophore transporters with different substrate specificities have evolved, e.g. Sit1 homologs for hydroxamate-type fungal siderophores in *C. glabrata* (Nevitt and Thiele 2011), *C. albicans* (Heymann *et al*. 2002; Lesuisse *et al*. 2002), *Cr. neoformans* (Tangen *et al*. 2007) and *S. cerevisiae* [Arn1–4, with Arn3 and Arn4 specific for bacterial ferroxamines and Enterobactin B, respectively (Heymann, Ernst and Winkelmann 2000a,b; Yun *et al*. 2000)] and in many other fungi. *Candida glabrata* Sit1 enhances fungal survival in macrophages (Nevitt and Thiele 2011), and *C. albicans* Sit1 is required for invasion of human epithelial cells *in vitro* (Heymann *et al*. 2002); in the absence of xenosiderophores, these observations seem puzzling, and although mammals were recently found to produce siderophores (Devireddy *et al*. 2010), these are similar to enterobactin und thus unlikely to be taken up via Sit1. Accordingly, *SIT1* deletion causes no attenuation in virulence of *C. albicans* in a systemic mouse model of infection (Hu *et al*. 2002). Similarly, *Cr. neoformans* Sit1 deletion mutants showed changes in melanin and capsule formation and in cell wall density, but were not reduced in virulence (Tangen *et al*. 2007)—however, there are six more potential siderophore transporters encoded in the *Cr. neoformans* genome (Jung and Kronstad 2008).

Overall, the ability to use a broad spectrum of xenosiderophores likely reflects microbial competition for iron. This would make such a strategy advantageous when close interspecies contacts are frequent, such as in biofilms in the oral cavity, gut or vagina, as well as generally in co-infections. However, in the absence of any evident producer, the role of xenosiderophore binding during dissemination in blood or host tissue remains unclear at best. In these environments, it seems more important that many fungi have developed multiple mechanisms to directly exploit iron-binding molecules of the host. *Candida albicans* shows an impressive versatility in using host sources and can directly or indirectly obtain iron from hemoglobin (Moors *et al*. 1992), hemin (Santos *et al*. 2003), ferritin (Almeida *et al*. 2008) and transferrin (Knight *et al*. 2005). Similarly, *Cr. neoformans* can use transferrin (Jung *et al*. 2008), heme and hemin (Jung *et al*. 2008; Cadieux *et al*. 2013; Hu *et al*. 2015), and *H. capsulatum* is known to obtain iron from transferrin and hemin (Timmerman and Woods 1999; Foster 2002), but *Aspergillus* spp. appear to be unable to acquire iron from heme (Vaknin *et al*. 2014).

In hemoglobin, iron is incorporated in heme in its ferrous form and can be acquired by *C. albicans* and *Cr. neoformans* with specific heme uptake mechanisms. The former relies on a family of heme receptors [Rbt51 (Moors *et al*. 1992; Weissman and Kornitzer 2004)] and hemophores [Rbt5, Pga7, Csa2 (Weissman and Kornitzer 2004; Weissman *et al*. 2008; Kuznets *et al*. 2014; Nasser *et al*. 2016)] for initial uptake followed by ESCRT complex-mediated internalization into the vacuole via the endocytic pathway (Weissman *et al*. 2008). In *Cr. neoformans*, the ESCRT complex similarly has a pronounced role in heme utilization [Vps23, Vps22, Snf7 (Hu *et al*. 2013, 2015)] along with the putative hemophore Cig1 (Cadieux *et al*. 2013). The internalized heme-bound iron is then released by a heme oxygenase, which has been described in many *Candida* species and in *S. cerevisiae* to recycle self-generated heme (Santos *et al*. 2003; Kim *et al*. 2006). Other host iron sources containing Fe^3+^ can also be taken up directly, or, more commonly, the bound Fe^3+^ is first extracted from host molecules (or siderophores) on the cell surface via ferric reductases. Fe^2+^ is then oxidized again by permease-coupled multicopper ferroxidases followed by trans-membrane transport of Fe^3+^ via high-affinity permeases to complete the uptake process. This system is especially important for virulence in non-siderophore producing fungi such as *Cr. neoformans* (Jung *et al*. 2009; Han, Do and Jung 2012), *C. albicans* (Ramanan and Wang 2000; Fang and Wang 2002; Knight *et al*. 2005; Cheng *et al*. 2013) and *C. glabrata* (Srivastava, Suneetha and Kaur 2014), which heavily rely on the reductive pathway for iron uptake to facilitate growth and virulence (Srivastava, Suneetha and Kaur 2014; Gerwien *et al*. 2016, 2017). In contrast, while *A. fumigatus* siderophore synthesis mutants were dramatically attenuated in virulence (Hissen *et al*. 2005), defects in reductive iron assimilation had no significant effect (Schrettl *et al*. 2004). Similarly, other siderophore producers, such as *Fusarium graminearum* (Greenshields *et al*. 2007) or *Al. brassicicola* (Oide *et al*. 2006), cannot fully compensate the loss of siderophore-mediated iron uptake by the reductive uptake system alone.

As described above, the reductive uptake system comprises reductase and linked permease/ferroxidase functions. Pathogenic fungi commonly have large families of cell-surface NAD(P)H-dependent ferric reductases at their disposal, such as *Cr. neoformans* (eight known reductases) (Saikia *et al*. 2014), *C. albicans* (18 putative) (Jeeves *et al*. 2011; Xu *et al*. 2014b) or *A. fumigatus* (15 putative) (Blatzer, Binder and Haas 2011)—with no number currently available for *H. capsulatum. Candida albicans* Fre2, Fre5/Frp1 and Fre9 (Bensen *et al*. 2004; Baek, Li and Davis 2008) are expressed under alkaline conditions, and there are indications that Fre2 might be secreted or shedded under azole treatment (Sorgo *et al*. 2011). In *Cr. neoformans*, transcription levels of Fre3 seem to be associated with virulence: RNAi suppression of *FRE3* decreased survival in macrophages, while artificial upregulation led to increased virulence in mice (Hu *et al*. 2014).

Ferric reductases are best characterized in *S. cerevisiae*, where, despite obvious redundancy, the nine known members each play specific roles in siderophore-Fe reduction (Fre1, Fre2, Fre3, Fre4) (Martins *et al*. 1998; Yun *et al*. 2001), copper reduction (Fre1, Fre2, Fre7) (Martins *et al*. 1998) and presumably in intracellular transmembrane shuttling at the vacuole (Fre6) (Huh *et al*. 2003). In *C. albicans*, similar specific functions have been attributed to Fre7 and Fre10 as cupric reductases (Jeeves *et al*. 2011). *Candida glabrata* (Srivastava, Suneetha and Kaur 2014) and the fission yeast *Schizosaccharomyces pombe* (Roman *et al*. 1993) are notable exceptions, since they each possess only two ferric reductase genes. In *C. glabrata*, the lack of *FRE6* has been associated with attenuated virulence in a *Drosophila* model (Brunke *et al*. 2015) and slightly decreased kidney fungal burdens in mice (Srivastava, Suneetha and Kaur 2014). However, our own work has shown that both Fre6 and Fre8 might have roles other than ferric or cupric reduction in *C. glabrata*, since this fungus does not exhibit evident surface ferric reductase activity (Gerwien *et al*. 2017). Finally, low-affinity broad-spectrum metal transporters for iron, copper and zinc have been identified in *S. cerevisiae* (Fet4) (Dix *et al*. 1994) and in *C. glabrata* (Fet4) (Srivastava, Suneetha and Kaur 2014; Gerwien *et al*. 2016) with possible orthologs in *Cr. neoformans* (Jacobson, Goodner and Nyhus 1998; Jung *et al*. 2008) and in *Sc. pombe* (Dainty *et al*. 2008).

Non-siderophore secreted molecules with the capacity to bind and reduce iron are also known in fungi. For instance, *H. capsulatum* uses the glutathione-dependent γ-glutamyltransferase Ggt1 to extracellularly reduce ferric iron from siderophores, transferrin and hemin (Timmerman and Woods 1999; Timmerman and Woods 2001; Zarnowski *et al*. 2008). Non-enzymatic ferric reductants are also excreted by this fungus (Timmerman and Woods 1999), although their exact nature is still unknown. In *Cr. neoformans*, 3-hydroxyanthranilate has been identified as an extracellular ferric reductant, but additional active compounds seem to exist (Nyhus, Wilborn and Jacobson 1997; Jacobson, Goodner and Nyhus 1998; Jung *et al*. 2008). As melanized *Cr. neoformans* cells reduce iron at a much higher rate than non-melanized cells, ferric reduction activity may be associated with this polymer (Nyhus, Wilborn and Jacobson 1997). In *S. cerevisiae*, excretion of anthranilate and 3-hydroxyanthranilate correlates with ferric reduction capacity in the extracellular medium, although, counterintuitively, cells grown in iron-rich medium show a higher secretion than those in iron-poor medium (Lesuisse *et al*. 1992). Likewise, culture supernatants of *C. albicans*, *C. glabrata* and *S. cerevisiae* show ferric reduction activity, which depends on a so far unknown low-molecular-weight compound (Gerwien *et al*. 2017), and *A. terreus* has recently been shown to secrete terrein under iron starvation, which acts as a ferric reductant and can partially rescue strains defective in siderophore biosynthesis (Gressler *et al*. 2015).

In a similar fashion, the active lowering of the environmental pH can increase iron bioavailability, either by increasing the overall solubility or via pH-dependent release of iron from host molecules such as transferrin (Lestas 1976). *Histoplasma capsulatum* is known to exploit this strategy inside macrophages, keeping the intraphagosomal pH at 6.5 (Eissenberg, Goldman and Schlesinger 1993). This is alkaline enough to inhibit phagolysosome function, but acidic enough to keep iron accessible and possibly even release it from host transferrin (Newman *et al*. 1994; Hilty, Smulian and Newman 2008). In fact, this strategy was found to be essential for intracellular growth and virulence of *H. capsulatum* (Hilty, Smulian and Newman 2008). Similar mechanisms are probably also used by other fungi with the ability to manipulate phagosomal pH, like, for example, *C. glabrata* (Kasper *et al*. 2014).

Excess iron is stored both as a stockpile for times of need and to avoid its toxicity at high concentrations. Storage is mediated either by vacuolar polyphosphates or by intracellular siderophores (see above); with the exception of zygomycetes, ferritin-like molecules with this purpose are so far unknown in fungi (Carrano, Bohnke and Matzanke 1996). In *S. cerevisiae*, the transporter Ccc1 mediates vacuolar iron (and manganese) import (Lapinskas, Lin and Culotta 1996; Li *et al*. 2001), while export is controlled by Smf3 (Portnoy, Liu and Culotta 2000) or a complex consisting of Fth1/Fet5 coupled to a ferric reductase, resembling the reductive uptake system of the plasma membrane (Urbanowski and Piper 1999). Ccc1 orthologs with similar roles in iron storage exist in *C. glabrata* (Gerwien *et al*. 2016), *C. albicans* (Xu *et al*. 2014a), *A. fumigatus* (Gsaller *et al*. 2012), *A. nidulans* (Eisendle *et al*. 2006) and *Sc. pombe* (Mercier, Pelletier and Labbe 2006), indicating that vacuolar iron storage is important in both siderophore producers and non-producers. Similarly, Smf3 has been associated with intracellular iron homeostasis in *S. cerevisiae* (Portnoy, Jensen and Culotta 2002) and in *C. albicans* (Xu *et al*. 2014a), and an ortholog is present in *C. glabrata*. Deletion of *C. glabrata* Fth1 or Fet5 does not cause sensitivity to the iron chelator bathophenanthroline disulfonate (Srivastava, Suneetha and Kaur 2014), although *FTH1* was found to be iron regulated (Gerwien *et al*. 2016). *Aspergillus nidulans* and *Sc. pombe* finally lack orthologs for both genes—however, in the latter, Abc3 has been suggested to have a similar role in vacuolar iron mobilization (Pouliot *et al*. 2010).

The organelles with the highest need for iron are mitochondria. Here iron-sulfur (Fe-S) clusters are synthesized as prosthetic group for respiratory chain complexes, the TCA cycle and various other metabolic processes. Consequently, a highly conserved short-term storage molecule has evolved in fungi and mammals: the mitochondrial matrix iron chaperone Yfh1 (Huynen *et al*. 2001), which has been found in *S. cerevisiae* (Babcock *et al*. 1997; Wilson and Roof 1997), *Sc. pombe* (Fxn1) (Wang *et al*. 2014), *C. albicans* (Santos *et al*. 2004) and *C. glabrata* (Srivastava, Suneetha and Kaur 2014).

### Iron-sensing and transcriptional regulation

Regulation of fungal iron homeostasis has mostly been studied in the model yeast *S. cerevisiae*. However, baker's yeast is barely representative of other fungi, since it employs a rather unusual regulation system, which among the pathogenic fungi has so far only been found in the closely related *C. glabrata* (Gerwien *et al*. 2016). In both species, an Aft transcription activator (Aft1 and Aft2 in *S. cerevisiae*) upregulates genes involved in Fe uptake under iron limitation (Yamaguchi-Iwai, Dancis and Klausner 1995; Ueta *et al*. 2012). Mechanistically, this is mediated by Fe-S clusters produced in the mitochondria, which—when present—bind the glutaredoxins Grx3 and Grx4 and enable them to interact with Aft1 to remove it from its promoter targets (Rutherford *et al*. 2005; Ueta *et al*. 2012). Such Fe-S clusters also play a role in adaptation to high iron, as they can activate the high iron-responsive regulator Yap5 (Li *et al*. 2012). Its limited range of target genes includes *CCC1* (Li *et al*. 2008), coding for the vacuolar iron importer (Li *et al*. 2001), and *CUP1* (Pimentel *et al*. 2012), encoding a copper-binding MT.

During Fe starvation, iron-requiring processes are post-transcriptionally further downregulated via degradation of mRNAs that carry the target sequence 5^΄^-(U)UAUUUAU(U)-3^΄^ in their 3^΄^UTR region. This process is mediated by the combined action of the RNA-binding proteins Cth1 and Cth2 in *S. cerevisiae* (Shakoury-Elizeh *et al*. 2004; Puig, Askeland and Thiele 2005; Puig, Vergara and Thiele 2008; Martinez-Pastor *et al*. 2013) and by a single Cth2 ortholog in *C. glabrata* (Gerwien *et al*. 2016). Thus, *C. glabrata* and *S. cerevisiae* (and likely their closest relatives) uniquely share the Aft/Cth iron regulatory system, although their opportunistic pathogenic and environmental lifestyles would at first glance suggest the need for vastly different iron homeostasis mechanisms. Interestingly, further Aft orthologs with roles in iron homeostasis have been identified in *Kluyveromyces lactis* (Conde e Silva *et al*. 2009), also a part of the *Saccharomycetaceae* clade (Gabaldon *et al*. 2013), and surprisingly in the evolutionary more distant yeast *C. albicans* (Liang *et al*. 2010; Xu *et al*. 2013). However, *K. lactis* lacks any Cth2 ortholog, whereas the one present in *C. albicans* (Zfs1) has no function in iron homeostasis, but influences biofilm formation (Wells *et al*. 2015). Notably also, *C. albicans* Aft2 has only a very minor function in iron homeostasis regulation (Liang *et al*. 2010; Xu *et al*. 2013), since, like most other fungi, *C. albicans* relies on a different iron regulation strategy.

This other system has so far been found (often with slight variations) in *C. albicans*, *Cr. neoformans*, both *A. fumigatus* and *A. nidulans*, and *Sc. pombe*. It usually comprises two repressors: a GATA transcription factor for the downregulation of iron acquisition (called Sfu1, Cir1, SreA, or Fep1 in these fungi) and a CCAAT-binding complex to downregulate iron consumption pathways (Hap43, HapX, HapX, or Php4) (Haas *et al*. 1999; Oberegger *et al*. 2001; Tuncher *et al*. 2005; Mercier, Pelletier and Labbe 2006; Hortschansky *et al*. 2007; Schrettl *et al*. 2008; Jung *et al*. 2010; Schrettl *et al*. 2010; Chen *et al*. 2011; Hsu, Yang and Lan 2011; Kronstad, Hu and Jung 2013). In *H. capsulatum* (Hwang *et al*. 2012), *N. crassa* (Zhou, Haas and Marzluf 1998), *Penicillium chrysogenum* (Haas, Angermayr and Stoffler 1997) and *U. maydis* (Voisard *et al*. 1993), a GATA factor (Sre1, Sre, SreP, Urbs1) with an iron-regulatory function has been characterized, but in these fungi, a complete iron-related CCAAT-binding complex has not been described yet. It is likely to be present, though, as both components play complementary roles for the efficient adaption to varying iron levels: under iron depletion, which is frequently encountered during active infections, the CCAAT-binding complex represses the iron-consuming cellular processes. At the same time, it indirectly induces iron acquisition by repressing the GATA transcription factor to alleviate its repressive effect on iron uptake. The latter function of the GATA transcription factor is in turn important under iron-replete conditions likely encountered by *C. albicans* cells commensally growing in the mammalian gut (Chen *et al*. 2011). In these environments, it also downregulates the CCAAT-binding complex, increasing the iron-consuming cellular processes. In *A. fumigatus*, HapX was recently shown to be important under both iron starvation and excess. Through different domains, this factor can either repress consumption or activate vacuolar sequestration of iron, depending on the current concentration of the metal (Gsaller *et al*. 2014). With these central roles, it is not surprising that a deletion of the CCAAT-binding complex results in attenuated virulence in *Cr. neoformans* (Jung *et al*. 2010), *C. albicans* (Hsu, Yang and Lan 2011; Singh *et al*. 2011) and *A. fumigatus* (Schrettl *et al*. 2010). Deletion of the GATA transcription factor leads to more varied outcomes, from complete avirulence in *Cr. neoformans* (Jung *et al*. 2006) to unchanged, wild-type level virulence in mouse infections for *A. fumigatus* Δ*sreA* (Schrettl *et al*. 2008) and *C. albicans sfu1*Δ/Δ (Chen *et al*. 2011). Notably, however, the *C. albicans sfu1*Δ/Δ mutant is severely defective in GI tract colonization, where iron is abundant (Chen *et al*. 2011).

*Candida albicans* adds a twist to this established system, as this fungus has incorporated a third regulator into the GATA/CCAAT partnership. Sef1 is an activator of Hap43 expression (Chen *et al*. 2011) and is required for full virulence (Chen and Noble 2012). Possibly, the two lifestyles of *C. albicans*—both as a pathogen and as a commensal in the gut where iron levels can change rapidly through food intake and microbial competition—require an additional stabilizing element in iron homeostasis regulation (Chen *et al*. 2011). Interestingly, a Sef1 ortholog is also present in *C. glabrata*, like *C. albicans* a commensal of mucosal surfaces, but with a vastly different regulatory network, and this has been shown to play an (albeit less pronounced) role in iron homeostasis (Gerwien *et al*. 2016).

Finally, with the close connection between pH and metal solubility, some fungi, such as *C. albicans* and *Cr. neoformans*, use the pH-responsive factor Rim101 to detect alkaline pH as a marker for iron starvation and signal to upregulate the iron acquisition systems (Bensen *et al*. 2004). Consequently, a *C. albicans rim101*Δ/Δ mutant is attenuated in virulence (Davis *et al*. 2000). Similarly, a *Cr. neoformans rim101*Δ/Δ mutant is unable to utilize heme (Cadieux *et al*. 2013), but was found to be hypervirulent (O’Meara *et al*. 2010) likely because of an (unrelated) defective shedding of capsule polysaccharides, which results in a hyperactivation of the host immune response (O’Meara *et al*. 2010, 2013).

The evolution of these various and partly redundant systems for iron homeostasis throughout fungal pathogens reflects the importance of this particular micronutrient. Adaptations occurred in response to host-induced scarcity, to conditions of varying pH, and to the changing availability of host iron sources. Our current knowledge on these adaptations is already being used to develop new therapeutic approaches, for example, by supporting the host in its iron restriction during fungal infections (reviewed in Bruhn and Spellberg 2015; Lamb 2015) or by using fungal iron acquisition systems as targets for potential vaccines—as has been done with *C. albicans* Als3, although its involvement in iron uptake was not known at that time (Spellberg *et al*. 2006, 2008). It is therefore noteworthy that, beyond well-researched examples such as *C. albicans* or *A. fumigatus*, many fungal iron acquisition strategies are likely still unknown to us.

## ZINC

Zinc is a structural and catalytic co-factor for many proteins, including the ubiquitous zinc finger DNA-binding proteins. Recently, zinc was also shown to be an intracellular second messenger in various transduction signaling pathways (Yamasaki *et al*. 2007). In fact, zinc is the second most abundant trace metal in the human body: there are more than 300 zinc-dependent enzymes, and ≈10% of human genes code for zinc-binding proteins (Andreini *et al*. 2006a). The importance of zinc is sadly evident in the two billion people who suffer from zinc deficiency, especially in developing countries: A lack of zinc leads to thymic atrophy and lymphopenia, and weakens both the innate and adaptive immune responses: phagocytosis, cytokine production by macrophages, host defense by neutrophils and natural killer cells, and antibody secretion of both T and B cells are all impaired under zinc deficiency (reviewed in Prasad 2012).

Like for humans, zinc is of high importance for microorganisms. Within the fungi, zinc homeostasis has been investigated mainly in *S. cerevisiae*: Following the pattern of a high proportion of zinc-binding proteins in eukaryotes, about 8% of the yeast proteome is thought to bind zinc (Andreini *et al*. 2006b) and more than 400 yeast genes are involved in growth under zinc limitation (North *et al*. 2012). These include genes essential for zinc homeostasis, but also endoplasmic reticulum (ER) function, oxidative stress resistance, protein folding, vesicular trafficking and chromatin modification. Moreover, SODs, which are essential for the detoxification of reactive oxygen species (ROS) generated by host cells, are copper-, manganese- and zinc-dependent enzymes (Huang *et al*. 2009).

Consequently, zinc is vital for growth and metabolism in both the host and pathogens. Thus, like for iron, there is a constant competition for zinc during infections, and zinc sequestration is another aspect of the vertebrates' nutritional immunity (Corbin *et al*. 2008). The frequently near-neutral pH in the host lowers the solubility of zinc and therefore restricts its accessibility for microorganisms. In the oral cavity, antimicrobial peptides within saliva, the histatins, are able to bind zinc and copper, which adds to their inhibitory effect on the growth of *C. albicans* (Gusman *et al*. 2001). Intracellularly, stimulated T cells, macrophages and dendritic cells decrease their lysosomal zinc content via the expression of the zinc transporter ZIP8, inducing zinc limitation for pathogens in the phagolysosome (Begum *et al*. 2002; Aydemir *et al*. 2009). Similarly, stimulated dendritic cells reduce their cytoplasmic zinc concentration by upregulating zinc exporters and downregulating zinc importers (Kitamura *et al*. 2006). Cytokine-activated macrophages restrict the intracellular growth of *H. capsulatum* by diminishing intracellular zinc availability (Winters *et al*. 2010) via binding to MTs and by sequestering labile zinc into the Golgi apparatus (Vignesh *et al*. 2013).

The host protein calprotectin inhibits bacterial and fungal growth by chelating transition metals, including zinc (Lulloff, Hahn and Sohnle 2004; Corbin *et al*. 2008). In fact, calprotectin is the most abundant cytosolic protein of neutrophils and is released mainly during the formation of neutrophil extracellular traps (NETs) as their key antifungal effector (Urban *et al*. 2009; Bianchi *et al*. 2011). *In vitro* the stimulation of neutrophils with phorbol myristate acetate triggers NET formation, which leads to the reduction of the supernatant zinc content, while no changes were detected for Fe, Cu and Mn concentrations (Niemiec *et al*. 2015). NET-dependent inhibition of fungal growth is consequently reversible *in vitro* by zinc supplementation (Urban *et al*. 2009; McCormick *et al*. 2010; Bianchi *et al*. 2011).

Fungi have developed sophisticated countermeasures to this host-imposed zinc limitation, including the expression of high-affinity membrane zinc importers and specialized secreted zinc uptake proteins, known as zincophores, in order to obtain zinc from the host environment (Citiulo *et al*. 2012). However, excessive zinc levels can also be toxic for cells—mainly due to competition with other metals for metal-binding sites in enzymes (McDevitt *et al*. 2011; Gu and Imlay 2013), as zinc does not participate in Fenton chemistry. Vertebrates use this to their advantage and are able to accumulate zinc to toxic levels in certain niches. As an example from bacteria, a drastic increase of the intraphagosomal zinc level leads to an impaired growth of *Mycobacterium tuberculosis*, although the bacterium can partially cope with this metal excess by the expression of metal efflux ATPases (Botella *et al*. 2011).

### Zinc homeostasis and uptake

**Figure 2.**
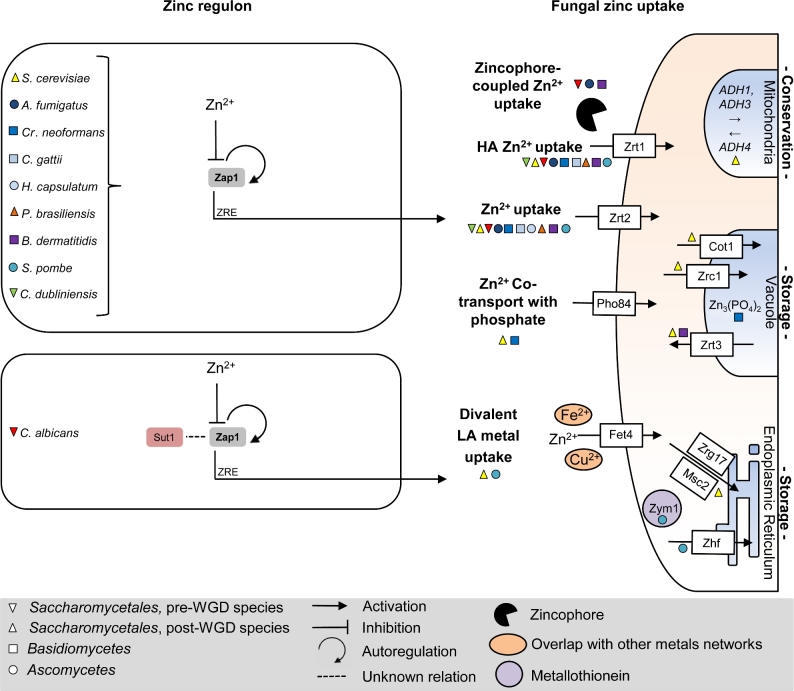
Fungal zinc homeostasis. Regulation of zinc homeostasis (left panel side) is shown for different fungal species (species is color coded, shape defines phylogenetic ancestry according to Gabaldon *et al*. 2013). Major transcription factors upregulated during zinc starvation to initiate fungal zinc uptake (right panel side) are written in bold. Orthologs are color shaded and aligned vertically. ZRE, recognition of target genes via zinc responsive elements. HA, high affinity; LA, low affinity.

Our knowledge of zinc transporters, their transcriptional regulation and zinc trafficking mechanisms within the cell (Fig. 2) is (again) based, for a good part, on studies in *S. cerevisiae*—all these were first described in baker's yeast. There are two known classes of eukaryotic zinc transporters: ZRT-IRT-like proteins (ZIP) (Grotz *et al*. 1998), which include *S. cerevisiae* Zrt1, Zrt2 and Zrt3 (MacDiarmid, Gaither and Eide 2000), and the cation diffusion facilitators (Paulsen and Saier 1997), represented by Zrc1, Cot1, Msc2 (Li and Kaplan 2001) and Zrg17 (Ellis, Macdiarmid and Eide 2005).

The uptake of zinc from the extracellular milieu takes place mainly via two ZIP transporters in *S. cerevisiae*, the high-affinity Zrt1 (Zhao and Eide 1996a) and the low-affinity Zrt2 membrane transporters (Zhao and Eide 1996b). Under severe zinc limitation, *ZRT1* expression increases 30-fold (Zhao and Eide 1996a) compared to optimal zinc conditions, while *ZRT2* is usually expressed only under mild zinc limitation. In addition, under conditions of low zinc, *S. cerevisiae* also expresses the low-affinity metal transporter Fet4 that imports zinc, iron and copper into the cell (Li and Kaplan 1998). An additional system that exists is the phosphate/H^+^ symporter family member Pho84, a known phosphate transporter, which is also able to import zinc complexed with phosphate (Jensen, Ajua-Alemanji and Culotta 2003).

*Aspergillus fumigatus* is able to robustly grow under a broader range of pH values than *S. cerevisiae*, especially in alkaline environments (Wheeler, Hurdman and Pitt 1991; Amich *et al*. 2010) where metal solubility is low (Martinez and Motto 2000). Of its eight putative ZIP transporters, ZrfA and ZrfB have functions in zinc uptake that resemble *S. cerevisiae* Zrt1, although ZrfB appears to be the main transporter (Vicentefranqueira *et al*. 2005). Interestingly, and in contrast to baker's yeast, this system is active only under acidic pH (Vicentefranqueira *et al*. 2005). In neutral to alkaline low zinc environments, resembling host tissue, *A. fumigatus* instead employs the ZrfC zinc transporter, which does not have a *S. cerevisiae* ortholog (Amich *et al*. 2010). Its ability to acquire zinc in alkaline environments seems to depend on its long N-terminus (not present in ZrfA and ZrfB), which contains four putative zinc-binding motifs (Amich *et al*. 2010). Consequently, this N-terminal sequence was found to be important for zinc uptake during lung infections, and it enables growth even in the presence of zinc-binding calprotectin (Amich *et al*. 2014).

The *Cr. neoformans* and *Cr. gattii* zinc uptake systems comprise the ZIP transporters Zip1 and Zip2, orthologs of *S. cerevisiae* Zrt1 and Zrt2, respectively (Do *et al*. 2016). In both fungi, the high-affinity membrane transporter Zip1 is the main (pH-independent) zinc importer, while Zip2 seems to contribute little, if anything, to zinc uptake *in vitro* (de Oliveira Schneider *et al*. 2015; Do *et al*. 2016). In *Cr. gattii*, both transporters must be deleted for a visible effect on virulence (de Oliveira Schneider *et al*. 2015), while in *Cr. neoformans*, deletion of Zip1 already results in attenuation in a mouse model of cryptococcosis (Do *et al*. 2016). However, residual virulence even in a *Cr. neoformans zip1*Δ*zip2*Δ double deletion mutant hints towards additional, still undetected zinc uptake mechanisms in this fungus and possibly, *Cr. gattii*. Interestingly, a connection between phosphate uptake and zinc homeostasis was shown for *Cr. neoformans* (Kretschmer *et al*. 2014), which could imply a role of its Pho84 homologs in zinc uptake similar to *S. cerevisiae*. Further zinc-regulated homologs of Zrt1 and/or Zrt2 have been described in *H. capsulatum* (Dade *et al*. 2016), *P. brasiliensis* (Parente *et al*. 2013) and *B. dermatitidis* (Muñoz *et al*. 2015), generally in connection to virulence—indicating the central role of zinc and this conserved acquisition system in fungal diseases.

Not surprisingly, *C. albicans* follows the same pattern of transport via Zrt1 and Zrt2 ZIPs (Kim *et al*. 2008), and again zinc uptake was found to be upregulated in the early stages of *C. albicans* infection in mice (Xu *et al*. 2015). However, the *C. albicans* zinc uptake system was shown to additionally include a ‘zincophore’ (Citiulo *et al*. 2012). In response to alkaline pH and to zinc limitation, *C. albicans* releases the metalloprotease-like Pra1 into the medium, where it is able to bind zinc ions with high affinity. Zinc-loaded Pra1 can then bind back to Zrt1, in a manner reminiscent of siderophores used by other fungi for iron (Citiulo *et al*. 2012). Interestingly, *PRA1* and *ZRT1* are co-expressed (Ihmels *et al*. 2005), as they share the same upstream intergenic region, and both were found to be upregulated on epithelial cells and in a liver infection model (Thewes *et al*. 2007; Zakikhany *et al*. 2007). So far, the *C. albicans* Pra1-Zrt1 pairing is the only proven zincophore system in fungi, but a similar locus structure is conserved in *A. fumigatus*: *ASPF2-ZRFC* is orthologous to *PRA1-ZRT1* (Amich *et al*. 2010), and like Pra1, AspF2 is secreted in high amounts during infections (Segurado *et al*. 1999). Not surprisingly, a possible zincophore function has recently been suggested (Amich *et al*. 2014). In *B. dermatitidis* mice infections, *BDFG_05357* is one of the most highly expressed genes. Like Pra1, it encodes an HRXXH domain-containing secreted protein, and has also been predicted to function as a zincophore (Muñoz *et al*. 2015). It seems that research into zincophores and their role in fungal pathogenesis is still gathering momentum.

High zinc levels can pose the opposite problem, and surplus zinc must be dealt with swiftly by the microorganism. In fungi, the vacuole serves as an organelle for zinc sequestration, storage and detoxification. Vacuolar zinc homeostasis has been investigated in some detail in *S. cerevisiae*, where it depends—among others—on the Zrc1 and Cot1 zinc importers of the vacuolar membrane (MacDiarmid, Gaither and Eide 2000). Surprisingly, *ZRC1* transcription is also induced under low zinc concentration, likely in anticipation of a possible sudden zinc excess: as all zinc importers are fully active, they will immediately relay any environmental increase in zinc abundance (MacDiarmid, Milanick and Eide 2003). Inside the vacuole, zinc is likely bound to polyphosphates, as shown for *Cr. neoformans* (Kretschmer *et al*. 2014). In contrast, *Sc. pombe* does not rely on the vacuole as a zinc sink; instead, the zinc homeostasis factor, Zhf, transports excess zinc into the ER (Borrelly *et al*. 2002; Clemens *et al*. 2002)—a function derived maybe from its *S. cerevisiae* counterpart, Msc2, which in a heterodimer with Zrg17 imports zinc into the ER for proper protein processing (Li and Kaplan 2001; Ellis *et al*. 2004). *Schizosaccharomyces pombe* strikingly also uses the metallothionein Zym1 to sequester zinc, similar to higher eukaryotes, but in contrast to other fungi, where MTs mainly sequester copper (Borrelly *et al*. 2002).

The vacuole not only serves as an emergency disposal site, but can also replenish cellular zinc in times of need. Zinc mobilization under starvation occurs via the Zrt3 vacuole zinc exporter in *S. cerevisiae* (MacDiarmid, Gaither and Eide 2000). Its orthologs have been found upregulated during co-incubation of *B. dermatitidis* with macrophages (Muñoz *et al*. 2015) and during zinc starvation in *C. dubliniensis* (Böttcher *et al*. 2015). Another approach to deal with low zinc is to conserve the metal by decreasing its use. *S. cerevisiae* reduces the expression of major zinc-dependent enzymes and induces expression of alternative proteins of identical function, which either require less zinc or different metals. For example, the alcohol dehydrogenases Adh1 and Adh3 (which bind two zinc ions each) are replaced under zinc limitation by Adh4, which only requires one zinc ion, allowing cells to continue fermentation even under zinc deficiency (Bird *et al*. 2006). Important infection-associated extracellular SODs of *C. albicans* (Sod4–6) and *H. capsulatum* (Sod3) uniquely use a single copper instead of the otherwise nearly universal Cu and Zn cofactors of SODs, likely reflecting the copper-rich, zinc-poor host environment (Gleason *et al*. 2014a)—a factor we will come back to in the section on copper.

### Zinc sensing and transcriptional regulation

In contrast to iron and copper, zinc is a redox-inactive metal and does not damage cells via ROS. However, it avidly binds to many metallation sites of proteins, replacing the native metal and interfering with their function. Hence, like for the other metals, zinc homeostasis must be precisely regulated. In yeast, the zinc responsive activator protein 1 (Zap1) is the major transcription factor regulating zinc homeostasis genes (Zhao and Eide 1997). It binds to conserved zinc responsive elements in the promoters of more than 80 genes, including *ZRT1, ZRT2, ZRT3, FET4* and *ZRC1* (Wu *et al*. 2008). Moreover, Zap1 positively autoregulates its own expression to ensure a robust response to zinc limitation (Zhao and Eide 1997; Wu *et al*. 2008). The structure of Zap1 was analyzed in detail in *S. cerevisiae*: it contains two activation domains, AD1 and AD2, which are evolutionary conserved within the fungal species (Frey and Eide 2011); AD1 is responsible for the induction of most Zap1 target genes, while AD2 regulates genes when zinc deficiency appears in concert with other stresses (Frey and Eide 2011). The intracellular zinc level is sensed via direct interaction of metal and protein: under a sufficient cytosolic zinc concentration, zinc ions directly bind AD1 and AD2 to inhibit the expression of Zap1 targets (Frey and Eide 2011). Overall, this system is highly conserved within fungi and can be found with few variations throughout the non-pathogenic and pathogenic species, including *Cr. gattii* (Zap1, de Oliveira Schneider *et al*. 2012) and *A. fumigatus* (ZafA, Moreno *et al*. 2007), and in both it was found important for full virulence.

For a fast downregulation of the importers during zinc repletion, post-translational effects come into play. Zrt1 is a stable membrane protein under low environmental zinc levels; however, the presence of zinc leads to its rapid ubiquitination and internalization for vacuolar degradation (Gitan *et al*. 1998). Moreover, under low zinc, Zap1 activates the expression of *PIS1*, encoding a phosphatidylinositol synthase, and *DTT1*, encoding a diacylglycerol pyrophosphate phosphatase, which results in increased levels of phosphatidylinositol and decreased levels of phosphatidylethanolamine in the membrane (Carman and Han 2007). This change in the membrane phospholipid composition is thought to influence the function and the localization of membrane zinc transporters.

The *C. albicans* Zap1 ortholog, also called Csr1, controls zinc homeostasis including Pra1 expression (Nobile *et al*. 2009), but is, of note, also involved in filamentation and biofilm matrix elaboration (Kim *et al*. 2008; Nobile *et al*. 2009)—two important contributors to *C. albicans* virulence. However, the virulence defect of a *csr1*Δ mutant likely depends not only on these morphological effects, but also directly on defective zinc homeostasis in the host. In support of this, a *csr1*Δ mutant of the closely related species *C. dubliniensis* shows no such filamentation defects, but still exhibits reduced virulence (Böttcher *et al*. 2015). Interestingly, in *C. albicans*, an additional transcription factor, Sut1, was recently implicated in controlling Csr1 expression *in vivo*, but surprisingly not *in vitro* (Xu *et al*. 2015). No functional relationship between the two *S. cerevisiae* counterparts is known (Xu *et al*. 2015), which suggests that this seemingly host-specific interaction is an adaptation to the pathogenic lifestyle of *C. albicans*. It will be interesting to see whether any other pathogen exhibits a similar departure from *S. cerevisiae*’s zinc regulation template.

A final twist is the pH-dependency of zinc uptake. As mentioned before, *A. fumigatus* switches from zinc uptake via ZrfA and ZrfB to ZrfC (and possibly AspF2) depending on the environment's alkalinity. While the Zap1 ortholog ZafA activates all transporters under zinc limitation independent of pH, the pH-dependent transcription factor PacC represses ZrfA and ZrfB under alkaline pH (Amich, Leal and Calera 2010) and ZrfC/AspF2 under acidic conditions (Amich *et al*. 2010). In *C. albicans*, expression of the Zrt1/Pra1 zincophore is similarly alkaline specific via the PacC ortholog Rim101 (Bensen *et al*. 2004; Citiulo *et al*. 2012; Xu *et al*. 2015), mirroring the Rim101-dependent expression of iron uptake-related genes. It seems likely that these expression patterns evolved as highly effective systems to deal with the low solubility of metals under alkaline conditions.

## COPPER

**Figure 3.**
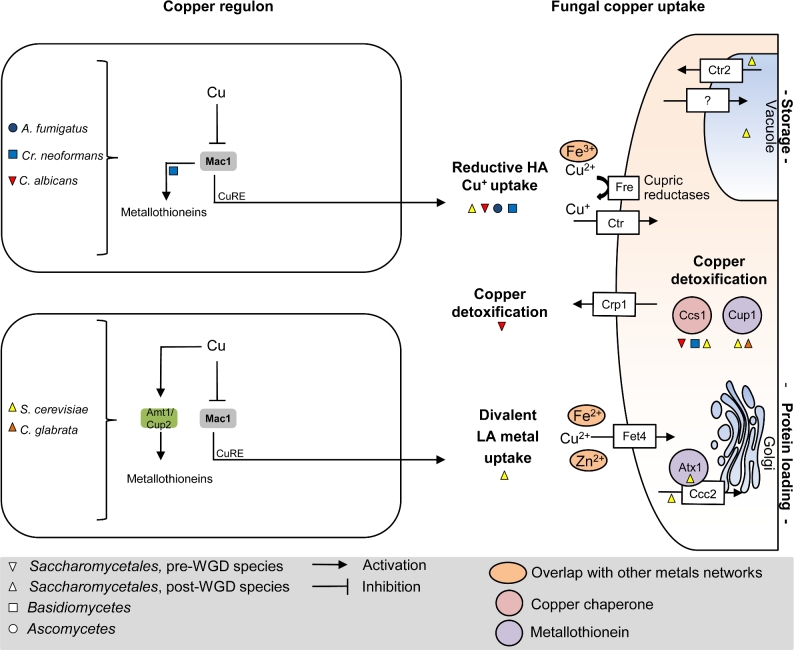
Fungal copper homeostasis. Regulation of copper homeostasis (left panel side) is shown for different fungal species (species is color coded, shape defines phylogenetic ancestry according to Gabaldon *et al*. 2013). Major transcription factors upregulated during copper starvation to initiate fungal copper uptake (right panel side) are written in bold. Orthologs are color shaded and aligned vertically. CuRE, recognition of target genes via copper responsive elements. HA, high affinity; LA, low affinity.

Copper is in many ways a different beast than iron or zinc (Fig. 3). Like those metals, it is required as an essential trace element in many biochemical reactions, but it rapidly becomes highly toxic at increased levels (reviewed in Festa and Thiele 2011). Copper started to be bioavailable at a large scale only after the great oxidation event ≈2.4 billion years ago, when earth's atmosphere became oxidizing. Eukaryotes, which evolved after these events, consequently harbor many more Cu-containing proteins than the more ancient bacteria (Dupont *et al*. 2010). For the same reason, many Cu-containing enzymes have oxygen-related functions. For instance, the mitochondrial cytochrome c oxidase requires Cu for its function in the respiratory electron transport chain. Cytoplasmic or cell-wall associated Cu/Zn-SODs (like their mostly mitochondrial manganese-dependent counterparts) can protect fungal cells from externally and internally generated oxidative stress. Again, the *C. albicans* SODs are unusual: *C. albicans* is the only known organism to contain both Cu/Zn- and Mn-SOD enzymes in the cytosol (Lamarre *et al*. 2001)—in addition to the Cu-only variety of extracellular SODs mentioned above. The Mn-dependent Sod3 is expressed to replace the Cu-dependent counterparts under copper starvation, for example during infections of the murine kidney (Li *et al*. 2015). This flexibility probably tells as much about the necessity of SODs for pathogens as about the diverse metal environments *C. albicans* is facing during infections. In addition, copper has an important helper role as a cofactor in multicopper ferroxidases to allow the uptake of iron via the reductive pathway (see above). Finally, it also has an important function as a cofactor of laccases and tyrosinases (Shaw and Kapica 1972; Williamson 1994), which are required for the biosynthesis of melanin—an important virulence factor of pigmented fungi.

However, due to its toxicity, copper has also been used as an antimicrobial agent for much of human civilization. As a fungicide against plant pathogens, it is part of the Bordeaux mixture used in vineyards, and copper surfaces show promise as a weapon against pathogens in hospitals (Casey *et al*. 2010). Part of its toxic effects derives from the ability of Cu^+^ (under anaerobic, reducing conditions) to disrupt Fe-S clusters (Macomber and Imlay 2009) and from its high capacity to displace other metals from their coordination sites, as, according to the Irving-Williams series, Cu^2+^ forms the most stable complexes of the divalent transition metals (Irving and Williams 1948). Furthermore, like iron, it can also readily form ROS by the Fenton reaction by Cu^+^/Cu^2+^ redox cycling under aerobic conditions, although the precise role of this for microbes is somewhat disputed (Macomber, Rensing and Imlay 2007), and in fact copper seems even more toxic under anaerobic than under aerobic conditions both for bacteria (Evans *et al*. 1986) and fungi like *S. cerevisiae* and *C. albicans* (Strain and Culotta 1996; Weissman, Shemer and Kornitzer 2000).

Given this comparatively high toxicity, the host and fungal strategies during infections differ significantly from the Fe-based nutritional immunity: instead of limiting access, the host seems to actively pump copper into microbe-containing phagosomes via the P-type ATPase ATP7A (Wagner *et al*. 2005; White *et al*. 2009). In fact, *Cr. neoformans* copper detoxification is activated during murine pulmonary infections, and the relevant MTs are required for virulence in this model (Ding *et al*. 2013). According to some reports, copper limitation may also play a role as an immune defense mechanism. A *Cr. neoformans* copper transporter was seen to be upregulated after phagocytosis by macrophage-like cells and during human cryptococcosis (Waterman *et al*. 2007, 2012), and the *C. albicans* copper transporter similarly shows upregulation upon phagocytosis (Lorenz, Bender and Fink 2004). Whether these observations represent a bona fide copper limitation or a loss of bioavailability due to the oxidative phagosomal environment (Waterman *et al*. 2007) remains to be seen. However, overlapping regulation of Cu uptake and resistance pathways (Ding *et al*. 2011), as well as possible confounding effects of the deletion and detection systems, seem to call for further investigation into the matter (Ding *et al*. 2013). Thus, the jury is still out whether both Cu ion overload and withholding are complementary strategies employed by the host, possibly depending on the microenvironment the fungus is facing.

### Copper homeostasis and uptake

Similar to iron, copper is usually reduced from Cu^2+^ to Cu^+^ (in part by the same cell-surface metalloreductases as for Fe) for efficient uptake and then imported via high-affinity Cu^+^ importers—Ctr1 in *C. albicans* (Marvin, Williams and Cashmore 2003), the functionally redundant Ctr1 and Ctr4 in *Cr. neoformans* (Ding *et al*. 2011), and at least two importers (CtrA2 and CtrC) in *A. fumigatus* (Park *et al*. 2014). In contrast to iron, no oxidase is involved in this process. In *S. cerevisiae* at least, the iron transporter Fet4 also imports copper with low affinity (Hassett *et al*. 2000). Another source of copper in addition to the surrounding medium is the vacuolar storage. In *S. cerevisiae*, the transmembrane copper transporter Ctr2, a homolog of Ctr1, allows copper mobilization from this organelle (Rees, Lee and Thiele 2004) with the help of a metalloreductase in the vacuolar membrane (Rees and Thiele 2007), mimicking the cytoplasmic membrane setup. Pathogenic fungi like *C. albicans* possess orthologs of these proteins, but their role in virulence has not been investigated so far.

Once intracellular, the potentially toxic Cu^+^ is immediately bound by different specific chaperones, which allow its quick and targeted transport to Cu-requiring enzymes. For example, Ccs1 proteins deliver copper to the Cu/Zn-SODs of *C. albicans* (Gleason *et al*. 2014b), *Cr. neoformans*, *S. cerevisiae* (Liu *et al*. 1997) and in fact nearly all eukaryotes (Leitch *et al*. 2009). Similarly, Atx1 homologs escort copper to Ccc2 Cu-transporting ATPases of the Golgi membrane (Lin *et al*. 1997; Huffman and O’Halloran 2000). These then pump the metal into late secretory vesicles to serve as a cofactor, for example, in the aforementioned Fe multicopper oxidases or laccases. This also intimately links copper to iron homeostasis, as multicopper oxidases are required for efficient iron uptake in fungi like yeast or *C. albicans* (Askwith *et al*. 1994; Eck *et al*. 1999; Cheng *et al*. 2013).

A similar binding mechanism prevents toxicity under high copper conditions. MTs, small proteins rich in cysteine residues, can sequester Cu (and, especially in non-fungal organisms, other metals) to render it biologically inactive. They are also present in plants and animals, but in very few bacteria—one example being specifically the pathogenic mycobacteria (Gold *et al*. 2008). Characteristically, the genes coding for MTs vary strongly in numbers between species: in pathogenic fungi, some *C. glabrata* strains harbor more than 30 copies of the MT-IIa gene, in addition to one copy each of MT-IIb and MT-I (Mehra, Garey and Winge 1990; Mehra *et al*. 1992). Similarly, *S. cerevisiae* can increase its copy number of the *CUP1* metallothionein gene and thereby obtain higher Cu resistance (Fogel and Welch 1982). No such mechanism has been described for *C. albicans* with its three known MTs or *C. neoformans* with its two (Ding *et al*. 2011) so far. Similarly, it seems that in *S. cerevisiae* copper is also detoxified, like other metals, via the vacuolar storage (Szczypka *et al*. 1997; Jo *et al*. 2008), but little is known about this process in other fungi.

In *S. cerevisiae* (and likely other fungi), high intracellular Cu levels furthermore rapidly block the Ctr1 Cu importer by direct binding and subsequent conformational changes to restrict copper influx (Wu *et al*. 2009). However, *C. albicans* achieves its high intrinsic Cu resistance (when compared to *S. cerevisiae*) also by active outward transport over the plasma membrane by Crp1, a P-type ATPase (Riggle and Kumamoto 2000; Weissman *et al*. 2000), in a process functionally resembling the copper transport by Ccc2 ATPase into the Golgi (Weissman, Shemer and Kornitzer 2002)—or even into the phagosome by the host's ATP7A, in an interesting example of a molecular-level arms race using the same mechanism on both sides. This export mechanism, although common in bacteria (reviewed in Samanovic *et al*. 2012) and present in other eukaryotes, has so far been found only in *C. albicans* and—very recently—in *A. nidulans* (Antsotegi-Uskola, Markina-Inarrairaegui and Ugalde 2017).

### Copper sensing and transcriptional regulation

Low copper levels lead to an activation of the transcription factor Mac1 in *S. cerevisiae* (Jungmann *et al*. 1993), and the same is true for its orthologs in *C. albicans* (Mac1; Marvin, Mason and Cashmore 2004), *A. fumigatus* (Afmac1; Kusuya *et al*. 2017) and most likely also *C. glabrata*. The Mac1 activator comprises a copper fist DNA-binding domain to recognize copper response elements, and a Cu-binding domain to gauge the intracellular copper concentration and inhibit DNA binding under copper replete conditions (Graden and Winge 1997). Under copper starvation, Mac1 binding leads to the expression of the dedicated copper transporter and metalloreductase genes via their upstream regulatory elements (Yamaguchi-Iwai *et al*. 1997). Under copper excess, Mac1 is quickly degraded to avoid copper toxicity (Zhu *et al*. 1998), and in contrast to copper-depleted conditions, *MAC1* mRNA exists in a readily degradable isoform when copper is present (Peccarelli *et al*. 2016). This Cu-dependent regulation directly influences virulence: deletion of the Mac1 ortholog Cuf1 reduces dissemination of *C. neoformans* to the mouse brain, and abolishes transcription of the copper-dependent laccase (Jiang *et al*. 2009). In *C. albicans*, Mac1 is—among other functions—responsible for shifting from the Cu-dependent Sod1 to the Cu-independent Sod3, by repressing the former and activating the latter (Li *et al*. 2015).

The *Cr. neoformans* Cuf1 (Lin *et al*. 2006; Waterman *et al*. 2007) is not only responsible for upregulation of copper uptake under starvation, but also positively regulates MTs under Cu excess (Ding *et al*. 2011). In fact, Cuf1 seems to be a hybrid factor, as in *C. glabrata* and *S. cerevisiae* these roles are separated, and in *C. glabrata* another transcription factor, called Amt1 [homologous to Cup2 or Ace1 in *S. cerevisiae* (Buchman *et al*. 1989; Szczypka and Thiele 1989)], is activated under high copper levels by the binding of four Cu^+^ ions to its N-terminal domain (Thorvaldsen *et al*. 1994). Active Amt1 then induces the transcription of all three MT genes and itself, leading to a positive autoregulatory loop and thus a robust copper resistance response (Zhou *et al*. 1992; Zhou and Thiele 1993; Koch *et al*. 2001). The role of its homolog in *C. albicans* is not well investigated so far (although it likely has similar functions), but the cAMP pathway has been implicated in copper resistance in this fungus. A deletion of *C. albicans GPA2* (encoding the G-protein α subunit upstream of protein kinase A) decreases Cu uptake, increases MT expression and hence renders the fungus more resistant to copper (Schwartz *et al*. 2013). Overall, the typical fungal response to high copper thus seems to be determined by a fast inactivation and degradation of the Mac1 activator homologs, and copper sequestration via upregulation of MTs by different mechanisms. However, our knowledge of these regulatory systems still lacks behind what we have learned about zinc and especially iron homeostasis in fungal pathogenesis.

## NICKEL

Nickel is a comparatively rare metal, but an efficient fungicide that seems to exert its effects mainly by interfering with the carbohydrate metabolism and DNA repair, by production of ROS (albeit less than copper or iron), and by membrane damage (reviewed in Macomber and Hausinger 2011). Many of these effects are exerted by nickel replacing the original metal in metalloenzymes—and as nickel is rather stable in the Ni^2+^ state, this replacement abolishes the redox function of the metal cofactor (Macomber and Hausinger 2011). At high external concentrations, nickel can non-specifically enter the microbial cell via the magnesium transport system. Still, dedicated uptake systems for this mostly toxic transition metal also exist, especially in bacteria (Zhang *et al*. 2009), and a functional Ni permease with high similarity to its bacterial co-family members has, for example, been found in *Sc. pombe* (Eitinger *et al*. 2000). So why would microbes, and especially fungi, actively import nickel? In *Sc. pombe*, this seems to be related to its urease activity (Eitinger *et al*. 2000), which requires Ni to allow the use of urea as a nitrogen source and the concomitant alkalization of the environment. For pathogens, ureases (and with them, most likely dedicated nickel permeases) often play important roles as virulence factors, for example, in *Coccidioides immitis* and in *Cr. neoformans* (Singh *et al*. 2013). With no known Ni metalloenzymes in vertebrates, nickel homeostasis has thus been suggested as a promising avenue for fighting infections (Morrow and Fraser 2013). However, the *Saccharomycetes*—like *S. cerevisiae*, *C. albicans* and *C. glabrata*—do not employ a Ni-requiring urease (Navarathna *et al*. 2010), and consequently seem to lack Ni permeases—instead, these fungi use non-nickel, biotin-requiring urea amidolyases to metabolize urea (Navarathna *et al*. 2010). In *A. fumigatus*, a nickel permease homolog can be found in the genome, but little is known so far about its potential role in virulence.

Excess nickel, as is so often the case with toxic metals, is sequestered into the vacuole by *S. cerevisiae* (Nishimura, Igarashi and Kakinuma 1998)—in this case with the help of the avid nickel binder, histidine (Pearce and Sherman 1999). It seems likely that pathogenic fungi have similar mechanisms at their disposal, paralleling the existence of nickel resistance mechanisms in many bacteria. Overall, however, little is currently known about the role of nickel in fungal pathogenesis, and we may yet be surprised by unexpected findings in the future.

## MANGANESE

Manganese is required in the function of polymerases, sugar transferases of the Golgi and of course for the Mn-SODs especially of the mitochondria (reviewed for baker's yeast in Reddi, Jensen and Culotta 2009). Its intracellular concentration has been shown to vary significantly, over nearly two orders of magnitude (Reddi, Jensen and Culotta 2009). One reason may be that—in contrast to most of the other metals described here—manganese acts as an anti-oxidant at high concentrations, rather than a ROS producer. In fact, at high intracellular concentrations Mn-containing complexes can take the role of SODs in certain bacteria and in yeast SOD deletion mutants (Reddi *et al*. 2009). Excessive levels are nonetheless toxic to yeasts leading to the induction of apoptosis (Liang and Zhou 2007).

External manganese is taken up in baker's yeast via the Nramp transporters, Smf1 and Smf2 (Supek *et al*. 1996; Cohen, Nelson and Nelson 2000; Portnoy, Liu and Culotta 2000), and a possible ortholog in *C. neoformans* has been described to transport Mn and other metals (Agranoff *et al*. 2005). It has been suggested that Smf1 is responsible for keeping up the intracellular Mn levels required for its anti-oxidant action, while Smf2 imports manganese for the Mn-requiring enzymes (Luk and Culotta 2001; Reddi *et al*. 2009). These transporters are continuously expressed and regulated mainly post-translationally, and at sufficiently high (physiological) Mn levels they are continually targeted for vacuolar degradation (Reddi *et al*. 2009). Furthermore and in a manner similar to zinc, high extracellular manganese can be imported by yeast in complex with phosphate via the Pho84 transmembrane transporter (Jensen, Ajua-Alemanji and Culotta 2003). Once inside the cell, it can then be transported by the Golgi P-type Ca^2+^/Mn^2+^ ATPase, Pmr1, to serve as a cofactor in the secretory pathway (Dürr *et al*. 1998). In fact, a Pmr1 homolog is required for full *C. albicans* virulence due to this cofactor role in glycosylation (Bates *et al*. 2005). Finally, in *S. cerevisiae* at least, excess manganese is excreted via the secretory pathway, but also sequestered to the vacuole (like iron via Ccc1; Li *et al*. 2001), and in *C. albicans* its complexation with polyphosphate has been shown (Ikeh *et al*. 2016). If and how manganese can leave the vacuole again is still an open question, as no dedicated exporter has been described so far.

Due to these biological functions, the host employs Mn starvation to fight bacteria and possibly fungi. Macrophage phagosomes are severely limited for manganese (Jabado *et al*. 2000), and the host-defense protein, calprotectin, chelates manganese in addition to zinc (Corbin *et al*. 2008) and—as shown recently—iron (Nakashige *et al*. 2015). *In vitro* at least, Mn chelation by calprotectin reduces growth of *A. fumigatus* (Amich *et al*. 2014; Clark *et al*. 2016), and Mn withdrawal may thus play a role in fungal infections—although in contrast to bacteria, the effects of manganese limitation on fungal virulence are probably eclipsed by the removal of zinc and iron. As with nickel, research into the role of manganese in fungi may yet reveal some unexpected connections to pathogenesis, as our knowledge so far is comparatively incomplete.

## CONCLUSIONS

Metals clearly play a central role during fungal pathogenesis. This is shown by the sheer number and diversification of the regulatory, uptake and detoxification systems in fungal pathogens, and of course by the host's many efforts to efficiently withhold metals. We seem to have a good concept of how iron and—with a few gaps—zinc are acquired by fungi during infections, but for many of the metals that are experimentally more difficult to address, our knowledge is still quite limited. The protection mechanisms against many metals with toxic effects are not well established, nor are the uptake systems for those which are required only in minute amounts—from cobalt to silver or cadmium. It seems likely that fungal research can learn a lot from the bacterial field, as even though the molecular mechanisms may differ, the basic problems the microbes are facing are essentially the same, and analogous solutions may have been found by both groups of pathogens.

Metal homeostasis also presents a largely untapped resource for potential treatment options. The natural response of the host already indicates the effectiveness of targeting the microbial requirement for metals. Strategies that may be worthwhile to follow in the future include a knowledge-guided combination of deprivation and excess: withholding one metal to induce a partially unspecific uptake response, which is exploited to introduce toxic levels of another. It seems that the immune system may already follow this strategy inside the phagosome, as described above e.g. for copper. Metal-based drugs were found highly effective against parasites like *Leishmania* spp. or *Plasmodium* spp. (reviewed in Navarro *et al*. 2010), and it seems at least possible that a similar approach may prove useful for fungi as well. We hope that with this review, we have enabled the reader to see the connections and similarities between metals and among fungi, maybe forming the kernel of a new hypothesis. The potential and the need for many more findings still exist in this growing field.
